# Akonni TruTip^®^ and Qiagen^®^ Methods for Extraction of Fetal Circulating DNA - Evaluation by Real-Time and Digital PCR

**DOI:** 10.1371/journal.pone.0073068

**Published:** 2013-08-06

**Authors:** Rebecca C. Holmberg, Alissa Gindlesperger, Tinsley Stokes, David Lopez, Lynn Hyman, Michelle Freed, Phil Belgrader, Jeanne Harvey, Zheng Li

**Affiliations:** 1 Akonni Biosystems, Inc., Frederick, Maryland, United States of America; 2 Novartis Vaccines and Diagnostics, Inc., Emeryville, California, United States of America; Tor Vergata University of Rome, Italy

## Abstract

Due to the low percentage of fetal DNA present in maternal plasma (< 10%) during early gestation, efficient extraction processes are required for successful downstream detection applications in non-invasive prenatal diagnostic testing. In this study, two extraction methods using similar chemistries but different workflows were compared for isolation efficiency and percent fetal DNA recovery. The Akonni Biosystems TruTip technology uses a binding matrix embedded in a pipette tip; the Circulating Nucleic Acids Kit from Qiagen employs a spin column approach. The TruTip method adds an extra step to decrease the recovery of DNA fragments larger than 600 bp from the sample to yield an overall higher percentage of smaller molecular weight DNA, effectively enriching for fetal DNA. In this evaluation, three separate extraction comparison studies were performed - a dilution series of fragmented DNA in plasma, a set of clinical maternal samples, and a blood collection tube time point study of maternal samples. Both extraction methods were found to efficiently extract small fragment DNA from large volumes of plasma. In the amended samples, the TruTip extraction method was ~15% less efficient with overall DNA recovery, but yielded an 87% increase in % fetal DNA relative to the Qiagen method. The average percent increase of fetal DNA of TruTip extracted samples compared to the Qiagen method was 55% for all sets of blinded clinical samples. A study comparing extraction efficiencies from whole blood samples incubated up to 48 hours prior to processing into plasma resulted in more consistent % fetal DNA recoveries using TruTip. The extracted products were tested on two detection platforms, quantitative real-time PCR and droplet digital PCR, and yielded similar results for both extraction methods.

## Introduction

Interest in non-invasive prenatal diagnostics (NIPD) or testing (NIPT) is growing rapidly due to its potential to supplement the standard prenatal diagnostic methods of amniocentesis or chorionic villus sampling, which carry significant health risks including fetal deformation and miscarriage [1]. Testing for genetic information in cell-free fetal DNA (cffDNA) present in the mother’s plasma only requires a simple blood draw [1]. Though this less invasive testing method offers a lower-risk approach to prenatal screening, the sample analyte presents many challenges requiring special processing techniques. First, the presence of cffDNA at low concentrations (≤ 10%) in maternal plasma early in pregnancy when diagnostic testing is most desirable [2,3] necessitates processing and concentration of large sample volumes to yield adequate amounts of DNA for analysis [4]. Scaling to these larger volumes can be challenging for typical isolation approaches involving spin columns, vacuum manifolds or magnetic beads. For example, improper distribution of magnetic beads throughout the sample or clogging of filter columns by lipids and other sample debris can compromise results; equipment limitations for these methods also typically restrict input volumes to 250 µL-1 mL in order to maintain a reasonable workflow. Second, cffDNA is present in maternal plasma in a high background of maternal circulating DNA [2]. This low ratio of fetal to maternal DNA creates challenges in accurately detecting copy number variations or aneuploidies [5]. Recent studies have demonstrated that the type of blood collection tube (BCT) used, as well as storage time from venipuncture to plasma processing, have a considerable impact on the amount of maternal DNA recovered from the plasma sample [4,6]. Additional lysis of maternal white blood cells can occur if samples are not processed expeditiously or with proper cell preservatives, thereby increasing the maternal DNA and resulting in an even lower percentage of fetal DNA.

Different approaches have been taken to separate the fetal and maternal DNA in order to increase the percentage of fetal DNA in an effort to improve the resolution. These efforts include treatment with formaldehyde [7], restriction enzyme digestion [8] and magnetic capture hybridization [9]. It has been shown that cffDNA fragments are typically shorter compared to maternal DNA [2,10] prompting exploration of enrichment through size separation. Though increases of cffDNA of up to 50% has been reported using time- and labor-intensive techniques such as gel electrophoresis [11], these methods are not practical for implementation in routine clinical testing.

Target-specific detection methods, including PCR (digital [12] or otherwise) and targeted sequencing, represent the most common current technologies for NIPT, while next-generation sequencing technology, which infers the entire fetal genome, is emerging as a feasible approach [13–15]. This enrichment in fetal DNA is valuable for these analytical platforms which detect and measure fetal aneuploidy in a background of normal maternal ploidy. Other applications for cell-free DNA analysis are also emerging, especially in the setting of cancer diagnostics [16] and transplant patient monitoring [17,18]. All of these technologies and applications require up-front purification and concentration of the sample to be analyzed, which stresses the importance of finding a method capable of efficient and reliable recovery of freely circulating DNA.

Previous studies have evaluated methods for cffDNA isolation, but have focused on RhD genotyping of the fetus later in gestation, an application which requires lower sample volumes to detect the fetal DNA because the targeted sequences are unique to the fetus [19–21]. In contrast, NIPT requires a large sample volume in order to obtain enough fetal DNA at a high enough fetal: maternal ratio to detect genetic disorders involving copy number variations during early gestation. At the time of this study, the only commercially available extraction kit for the isolation of cell-free DNA able to accommodate volumes of at least 5 mL was the manual Qiagen Circulating Nucleic Acid Kit, which uses silica filter spin columns [4]. This method was compared to the newly developed technology from Akonni Biosystems, the TruTip^®^ extraction method [22] that also uses the BOOM^®^ silica binding chemistry [23] for nucleic acid binding and elution. TruTip consists of a porous rigid silica monolith inserted into a pipette tip that connects to standard laboratory pipettors or automated liquid handling systems. The binding matrix has greater porosity than a typical silica filter, which effectively lowers the backpressure, allowing bidirectional flow and processing of viscous samples such as plasma or blood without the high force of a vacuum manifold or centrifuge. Multiple passes of the sample across the binding matrix allow for a longer target residence time, higher probability of binding, and the ability to process sample volumes larger than the volume of the pipette tip itself.

The purpose of this study was to evaluate the Akonni Biosystems TruTip extraction technology against the gold standard Qiagen spin column, for recovery of fetal DNA from maternal plasma. The two technologies were tested for their relative performance in processing spiked samples using fragmented DNA to simulate cell-free DNA components to assess absolute recoveries. Two additional studies were performed on clinically relevant samples, one including a set of blinded maternal samples and one consisting of early gestational samples processed at different times after blood collection. Both quantitative real-time PCR (qPCR) and droplet digital PCR (ddPCR) were used to detect and compare DNA recovery by both extraction methods.

## Materials and Methods

### Plasma and DNA Samples

Non-pregnant female plasma samples for dilution series studies were obtained from Bioreclamation (Westbury, NY). Purified genomic DNA was fragmented using a Covaris S220 sonicator (Covaris, Woburn, MA). Male genomic DNA (Promega, Madison, WI) was fragmented to an average of 150 bp (range from 50–400 bp), to simulate fetal circulating DNA. Female genomic DNA (Promega, Madison, WI) was fragmented to an average of 800 bp (range from 100–1600 bp) to simulate maternal circulating DNA. For the dilution series study, 5 mL of non-pregnant female plasma was spiked with 200 ng fragmented female DNA with additional fragmented male DNA at 100 ng, 30 ng, 10 ng, 3 ng, 1 ng or 0 ng. These concentrations reflect the anticipated concentrations of maternal and fetal DNA in NIPT applications [3]. Samples were processed in triplicate using Akonni’s TruTip extraction method or Qiagen’s Circulating Nucleic Acid Kit.

In order to determine the comparative extraction efficiency of the two methods on a large set of male fetal specimens, 19 maternal plasma samples were collected by AllCells (Emeryville, CA) or StemExpress (Placerville, CA) from subjects at 5.3 to 13.3 weeks of pregnancy (mean of 9.8 weeks). This study was conducted according to an institutional review board–approved protocol (BioMed IRB, San Diego, CA). All study participants gave written informed consent. Five to six tubes of whole blood were collected from each subject, in either Streck Cell-Free DNA™ Blood Collection Tube (BCT, Omaha, NE) or BD ACD Vacutainer® (Franklin Lakes, NJ). Tubes were pooled together and centrifuged twice, first at 800 x g and second at1600 x g for Set 1, or first at 1600 x g and second at 16000 x g for Set 2 (Table 1), to obtain roughly 20-25 mL of total plasma. Five mL of plasma was extracted using either the Akonni TruTip or the Qiagen Circulating DNA Kit method. For Set 2, a replicate extraction was performed using TruTip with 7 mL input volume; lysis buffer, proteinase K, and binding buffer volumes were scaled accordingly.

**Table 1:** Blinded Clinical Sample Extraction Results.

**Set 1**			**Total DNA**		**Fetal DNA**			
**Sample ID**	**Gest. Age**	**Extraction Method**	**Avg Total DNA (Ges/µL)**	**Std Dev**	**Avg Fetal DNA (Ges/µL)**	**Std Dev**	**% Fetal**	**% increase of TT over Q**
1092B	11.4	Q	139.46	7.31	1.34	0.11	0.96%	
		TT	53.45	3.87	0.67	0.08	1.25%	30.2%
122B	9	Q	203.35	5.83	2.07	0.23	1.02%	
		TT	78.62	7.46	1.7	0.19	2.16%	111.8%
123B	8	Q	137.98	13.63	1.87	0.31	1.35%	
		TT	59.05	4.3	1.2	0.15	2.04%	51.1%
1081B	8.1	Q	173.23	3.39	1.77	0.16	1.02%	
		TT	49.24	0.55	1.38	0.24	2.80%	174.5%
1085B	8	Q	178.18	6.87	5.92	0.32	3.32%	
		TT	64.37	3.28	4.3	0.47	6.68%	101.2%
116B	9	Q	177.17	15.05	7.67	0.74	4.33%	
		TT	112.63	5.61	6.28	0.6	5.57%	28.6%
115B	14	Q	56.6	4.93	2.81	0.27	4.97%	
		TT	24.14	2.17	1.62	0.13	6.71%	35.0%
1057B	8.1	Q	96.73	1.35	5.93	0.26	6.13%	
		TT	38.76	6.3	4.07	0.35	10.51%	71.5%
1064B	12	Q	68.45	6.36	4.98	0.34	7.27%	
		TT	39.7	3.87	4.2	0.54	10.57%	45.4%
121B	24	Q	154.82	15.2	12.9	0.53	8.33%	
		TT	67.77	7.75	11.62	0.37	17.15%	105.9%
1080B	10.5	Q	277	9.97	34.03	0.08	12.29%	
		TT	151.84	15.43	29.65	0.12	19.53%	58.9%
**Set 2**			**Total DNA**		**Fetal DNA**			
**Sample ID**	**Gestation Age**	**Extraction Method**	**Avg Total DNA (Ges/µL)**	**Std Dev**	**Avg Fetal DNA (Ges/µL)**	**Std Dev**	**% Fetal**	**% increase of TT over Q**
5247B	6.1	Q	60.65	7.07	0.49	0.21	0.81%	
		TT (5 mL)	31.18	1.37	0.35	0.07	1.12%	38.58%
		TT (7 mL)	40.09	3.68	0.44	0.06	1.10%	35.50%
5010B	5.3	Q	26.26	3.05	2.53	0.09	9.65%	
		TT (5 mL)	13.75	0.65	1.61	0.09	11.71%	21.34%
		TT (7 mL)	13.47	1.19	1.63	0.1	12.10%	25.40%
5407B	7.4	Q	30.88	2.82	3.4	0.59	11.01%	
		TT (5 mL)	14.99	0.7	2.18	0.17	14.54%	32.09%
		TT (7 mL)	12.36	1.58	1.7	0.3	13.75%	24.92%
5623B	7.6	Q	38.09	5.01	3.89	0.16	10.21%	
		TT (5 mL)	26.53	1.52	3.89	0.16	14.66%	43.61%
		TT (7 mL)	29.14	4.15	4.21	0.29	14.45%	41.50%
5935B	9.1	Q	65.82	4.55	6.71	0.36	10.20%	
		TT (5 mL)	29.95	4.05	4.67	0.6	15.59%	52.87%
		TT (7 mL)	38.42	1.81	4.81	0.98	12.52%	22.74%
5410B	13.3	Q	54.45	3.82	5.57	0.55	10.22%	
		TT (5 mL)	25.83	3.14	4.21	0.34	16.30%	59.48%
		TT (7 mL)	27.75	3.7	4.01	0.52	14.45%	41.39%
5253B	6.4	Q	40.63	1.11	5.19	0.58	12.78%	
		TT (5 mL)	20.46	1.79	3.15	0.46	15.40%	20.47%
		TT (7 mL)	10.58	0.39	1.74	0.13	16.45%	28.69%
5930B	8.2	Q	51.87	1.92	8.46	0.29	16.31%	
		TT (5 mL)	27.92	4.56	5.7	0.19	20.42%	25.17%
		TT (7 mL)	30.59	2.5	6.15	0.47	20.10%	23.27%

Table of qPCR results from comparison extractions of maternal plasma. Set 1 used spin protocol 800/1600 x g with plasma sample input = 5 mL. Set 2 used spin protocol 1600/16000 x g with plasma sample input = 5 mL (with a duplicate TT sample at 7 mL plasma input volume, reagents scaled accordingly). Q = Qiagen, TT =TruTip, Ges = genome equivalents.

In order to determine the effect of processing time lags on percent fetal DNA recovery, 29 specimens of 60 mL of maternal whole blood, from 8 to 9.4 weeks gestation, were procured from StemExpress in Streck Cell-Free DNA™ BCT (Omaha, NE). This study protocol was approved by BioMed Institutional Review Board and all study participants gave written informed consent. The study was designed to exhaust the Design for Six Sigma (DFSS) methodology by taking into account all noise factors, control factors, and critical function responses. Samples were incubated at room temperature for 0, 24 or 48 hours (20 mL at each time point) prior to preparation of plasma using the double centrifuge protocol, first spin at 1600 x g and second at16000 x g. Volumes of each sample processed are listed Table S1. Gender testing was performed using 1-2 mL of sample extracted using the Qiagen method. Only 13 out of the 29 specimens collected were from subjects carrying male fetuses (data not shown), and these were used for further study. A single aliquot of plasma from the 13 male fetal specimens was extracted using the Akonni TruTip and Qiagen Circulating DNA Kit methods at each timepoint.

### Extraction Methods

#### TruTip protocol

Twenty milliliter Rainin pipette tips were fitted with the LPT4.0 TruTip binding matrix. The TruTip was attached to a SciLogix Levo Plus Motorized Pipette Filler (Berlin, CT) with an adapter to fit the tip. Rainin 20 mL EDP3 pipettes were also considered, however the pipette did not have the force to pull the plasma sample through the TruTip. The enrichment and concentration step of the procedure was performed using a 1 mL LPT 4.0 TruTip with a Rainin EDP3 electronic pipette (either single-channel, or multichannel with adjustable spacer, Mettler-Toledo, Columbus, OH).

**Figure 1:**
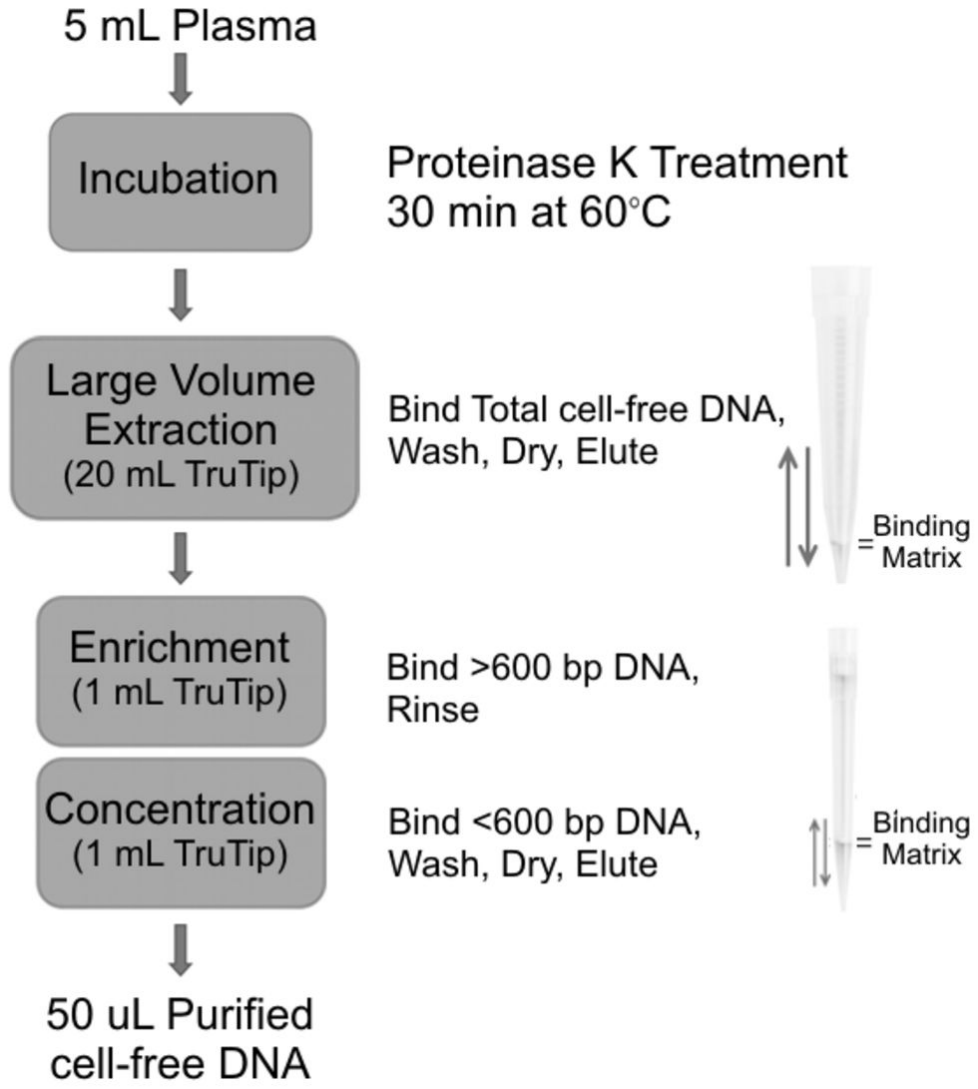
TruTip extraction workflow.

The optimized protocol, shown in Figure 1, was similar to that previously reported [24] with buffers supplied by Akonni Biosystems (cat 300-20541). Briefly, the method began with an initial incubation step of 5mL plasma for 30 minutes at 60° C, 615 µL proteinase K (Amresco, Solon, OH), 1 µg Carrier RNA (Life Technologies, Carlsbad, CA), and 6.2 mL Lysis Buffer CN-L1. After incubation, 12 mL Binding Buffer CN-B1 was added and mixed. Thereafter, a SciLogix Pipette Filler fitted with a 20 mL LPT4.0 TruTip was used to pass the sample/buffer mixture through the TruTip for 18 aspirate-dispense cycles to bind DNA to the TruTip matrix. The binding matrix was then washed with consecutive wash buffers (CN-W1, CN-W2, and CN-W4) of 2 mL volume for 1 cycle each to remove residual sample components and buffer salts that can serve as amplification inhibitors. The TruTip matrix was air-dried by cycling the pipettor 15 times in an empty tube. Finally, purified DNA was eluted from the TruTip matrix by cycling 250 µL of Elution Buffer A2 (pre-heated to 70° C) five times through the matrix. The elution step was repeated and volumes combined.

For the enrichment and concentration of fetal DNA, 495 µL of Binding Buffer CN-B2 was added to the final eluent from the 20 mL TruTip and briefly vortexed. A Rainin EDP3 pipette with a 1mL LPT4.0 TruTip was used to cycle the mixture 20 times across the TruTip to bind the larger nucleic acid fragments to the matrix. The matrix was then washed with 1.5 mL of Elution Buffer B for 5 cycles to rinse the larger fragments from the TruTip. Next, 575 µL of Binding Buffer CN-B3 was added to the sample tube and mixed. The sample mixture was cycled through the TruTip for 20 cycles to bind the remaining DNA to the matrix. The matrix was then washed with 700 µL of CN-W3 and CN-W4 for 1 cycle each to remove residual salts and aid in the evaporation of ethanol. Next, the matrix was dried by pipetting air through the tip for 15 cycles. Finally, the bound DNA was eluted by cycling 50 µL of Elution Buffer A2 through the TruTip 10 times. The eluted sample was used in PCR amplification as described below. The total elapsed time for this protocol was 1.5-2 hrs.

#### Qiagen protocol

The Qiagen Circulating Nucleic Acid Kit (Qiagen, Hilden, Germany) protocol was followed for processing 5 mL plasma samples. In brief, 500 µL proteinase K, 5 mL plasma, and 4 mL Qiagen buffer ACL were combined, vortexed, and incubated at 60° C for 30 minutes. Buffer ACB (9 mL) was added, vortexed, and incubated on ice for 5 minutes. The sample mixture was then added to the large volume tube extender attached to the spin column on the vacuum manifold. After the solution passed completely through the column, the bound DNA was washed by adding 600 µL Wash Buffer ACW1 to the column and pulled through by vacuum, followed by 750 µL ACW2 and finally 750µL 100% ethanol. The column was then removed from the vacuum manifold and centrifuged for 3 minutes at 20,000 x g rpm. Next, the column was placed in an open 2 mL microcentrifuge tube and heated at 56° C for 10 minutes in a heat block. To elute the DNA from the column, 50 µL of elution buffer AVE was added to the tube and incubated for 3 minutes at room temperature prior to a final centrifugation at 20,000 x g for 1 minute. The total elapsed time for this protocol was 2.5 hrs.

### Detection

A duplex PCR assay for both qPCR and ddPCR methods was used to quantitate the amount of fetal and total DNA isolated from the plasma samples. A multi-copy assay (~12-15 copies/genome) targeting the Y chromosome DYS-14 sequence was used to quantify the amount of male fetal DNA present in the eluted sample [25], and an assay targeting Chromosome 1 was used to quantify the total amount of DNA present [26]. Multi-copy assays have been shown to be more accurate than single-copy assays for assessing fetal DNA levels [27]. The reaction mixture contained 300 nM primers (IDT, Coralville, IA), 150 nM probes (IDT, Coralville, IA), and 1X Taqman Gene Expression Master Mix (Life Technologies, Carlsbad, CA) in a total volume of 20 µL, including 4 µL purified DNA. Thermocycling conditions for both qPCR and ddPCR methods were as follows: 50° C for 2 min, 95° C for 10 min, followed by 40 cycles of 95° C for 15 seconds, 60° C for 1 minute, and 72° C for 1 minute.

#### Real-time Quantitative PCR (qPCR)

Limits of detection were determined by real-time PCR using a LightCycler 480 system (Roche, Indianapolis, IN), while all maternal plasma sample extractions were run on an ABI StepOnePlus real-time system (Life Technologies, Carlsbad, CA). The same assay conditions described above were used for samples on both real-time systems. Three replicate qPCR tests were performed on each extracted sample. Data were analyzed using a fit point analysis method for the LightCycler480 and the threshold method for the StepOnePlus system. Total DNA and male fetal DNA concentrations and yields were calculated using the standard curve generated from serial dilutions of male genomic DNA (Promega, Madison, WI). Mean reported values are averages over all replicate qPCR assays per extraction.

#### Droplet Digital PCR (ddPCR)

Droplet digital PCR was performed using the QX100™ Droplet Digital™ PCR System from BioRad (Hercules, CA) according to the manufacturer’s instructions. Briefly, samples contained eluted DNA (<66 ng per 20 µL), 2X master mix, and 20X primer probe mix. Droplets were generated using the Droplet Generator (DG) with 70 µL DG Oil per well with a DG8 cartridge and cartridge holder, 20 µL PCR reaction mix, and DG8 gasket. Droplets were dispensed into the 96-well PCR plate by aspirating 40 µL from the DG8 cartridge into each well. The PCR plate was then heat-sealed with a foil seal and the sealed plate was placed in the PCR thermocycler. After the reaction, the droplets were read using the Droplet Reader, and QuantaSoft software converted the data into concentrations using Poisson distribution statistical analysis.

#### Analysis

Both qPCR and ddPCR data were analyzed and presented quantitatively as mean ± standard deviation (SD). Differences in extraction methods and data sets were evaluated by Students t-test (p-value) and correlations were calculated using the Pearson correlation coefficient (r). The reproducibility of different protocols was evaluated by coefficient of variation (CV). The effects of time delay on cell-free DNA recovery during blood sample processing were analyzed by one-way analysis of variance (ANOVA). JMP (SAS, Cary, NC) and R (GNU, freeware) software were used for statistical analyses.

## Results

### Dilution Series Comparison

**Figure 2:**
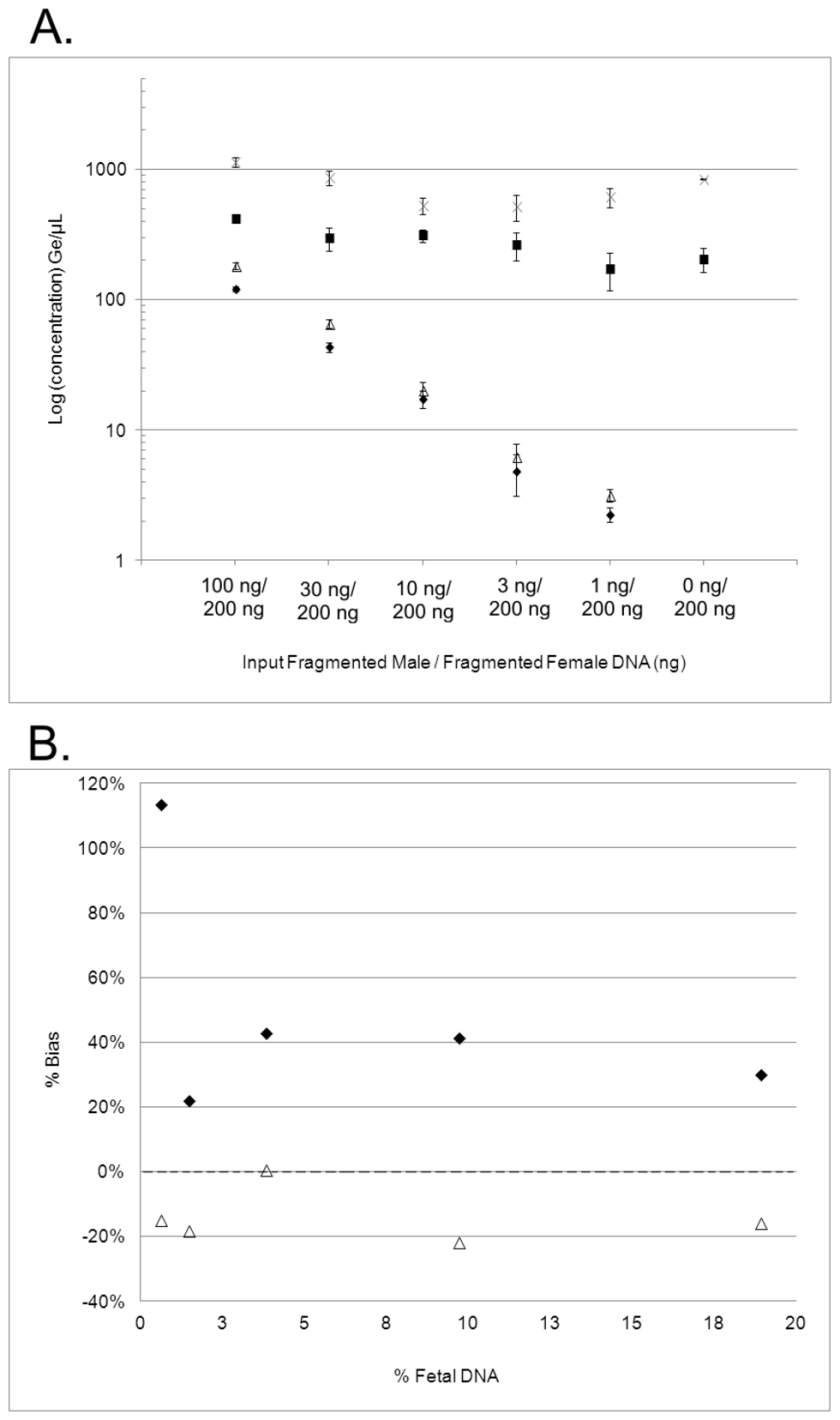
Amended Plasma Dilution Series Extraction Results. A) qPCR results from extracted samples containing fragmented male DNA ranging from 100 to 1 ng and total DNA including 200 ng fragmented female DNA per sample for TruTip (◊ and ■) and Qiagen (Δ and X) respectively, n=3 extractions each with n=3 per sample for PCR. Error bars indicate ± one standard deviation. B) Percent bias from actual % fetal DNA recovery for TruTip (◊) and Qiagen (Δ) over the same range of sample inputs (100-1 ng male and 200 ng female).

The recovery efficiency of fetal DNA in a background of maternal DNA was determined in the dilution experiment, where constant amounts of fragmented female DNA, and decreasing amounts of smaller fragmented male DNA, were amended into non-pregnant-female plasma. The dilution series study was designed around the amounts of fetal and maternal cell-free DNA typically found in plasma samples [3]. Male (fetal) DNA and total DNA concentrations (in genome equivalents (Ges) per µL) were plotted to compare the recovery of each over the range of input male fragmented DNA (from 100 ng to 1 ng) for both extraction methods (Figure 2A). The percent recovery of male fragmented DNA and total DNA were averaged over all inputs as shown in Figure 2A. Consistent yields were observed across the three replicates of each sample dilution, with % CVs of 15% and 19% (TruTip) and 13% and 15% (Qiagen) for male fragmented DNA and total DNA recovery, respectively. These results show comparable reproducibility for both methods. TruTip and Qiagen methods exhibited recoveries of 60% and 75% respectively for male fragmented DNA and 40.6% and 93.6% respectively for total DNA. Recoveries from both methods were higher for total nucleic acid recovery compared to those reported in previous studies using a modified QIAamp Blood Kit (~19%) or Triton/Heat/Phenol protocol (~39%) [28], suggesting both TruTip and Qiagen methods offer significant improvements over previously available protocols. The Pearson correlation value (r) comparing the TruTip and Qiagen elution concentrations over all input values was 0.9995 for male DNA (statistically insignificant with p = 0.09) indicating a strong correlation for the recovery of the male fragmented DNA. The results show that TruTip is equally efficient at isolating smaller fragments of DNA from large volumes of plasma. However, the correlation value was 0.5727 for total DNA (statistically significant with p = 0.001), suggesting a weak correlation between the methods for recovery of total DNA. This dissimilarity signifies the ability of the TruTip protocol to effectively suppress the recovery of larger fragments (maternal) compared to the Qiagen method, which indiscriminately isolates DNA of all sizes.

Figure 2B uses the data from the dilution series to compare the percentage of male DNA ([male DNA/total DNA] *100) recovered versus the actual % male DNA present in the sample, presented as % Bias. The latter was calculated using the quantitated real-time values of the input fragmented male DNA and the combination of input fragmented female DNA with the amount of cell-free female DNA from the plasma, 31.40 Ges/µL. The Qiagen % male recovery falls short in capturing a representative mixture of the analytes present in the sample, with an average 14% decrease compared to the actual percentage present in the sample. On the other hand, TruTip extraction resulted in an average 50% increase in percent male DNA recovery over the input range, and 84% increase compared to the Qiagen method. These results indicate the TruTip method’s ability to preferentially isolate smaller fragments of DNA from a mixed population sample.

### Performance with Clinical Samples

To verify that the enrichment effect observed in the spiked samples extracted by TruTip is applicable to isolation of cffDNA, the extraction methods were further compared using blinded clinical samples. The actual amounts of fetal and maternal DNA were unknown; thus, for all clinical samples the TruTip extraction efficiency was analyzed relative to the Qiagen method. A total of 19 specimens from subjects carrying male fetuses were extracted using both isolation methods and analyzed for fetal and total DNA recovery. Two centrifugation protocols were used to separate haematocytes from plasma. The lower setting protocol, 800/1600 x g yielded less plasma and required a significantly longer extraction time compared to the higher setting spin protocol, perhaps due to cell debris clogging the binding matrices in both extraction methods. The higher setting protocol, 1600/16K x g, yielded more than 15 mL total, enough volume to run a duplicate TruTip extraction.

Extraction results are shown in Table 1, arranged in order of decreasing % fetal DNA, with gestational age ranging from 5.3 to 13.3 weeks. The % fetal DNA recovery by TruTip was consistently higher, for all samples tested, than that by Qiagen, with an average percent increase of 74% for set 1 and 33% for set 2. The increases shown in both sets are statistically significant, with p values of 0.002 and 0.0003, respectively, for paired, single tailed t-test. The enrichment for set 1 is close to the percentage determined from the dilution series study of 84%. The lower enrichment of fetal DNA for set 2 suggests that the higher force spin protocol is better at removing unlysed maternal cells, thereby reducing the amount of maternal DNA in the isolated sample. Overall comparison of both clinical sample sets between the two isolation methods results in Pearson correlation values of 0.993 for fetal DNA recovery and 0.826 for total DNA recovery. This trend of equivalent fetal DNA recovery and lower total DNA recovery using the TruTip method as compared to Qiagen, mirrors the results from the dilution series study. Replicate extractions were performed for Set 2 using TruTip on 5 mL and 7 mL plasma samples with no statistically significant difference detected between replicates.

### Blood Collection Tube Time Point Study

**Figure 3:**
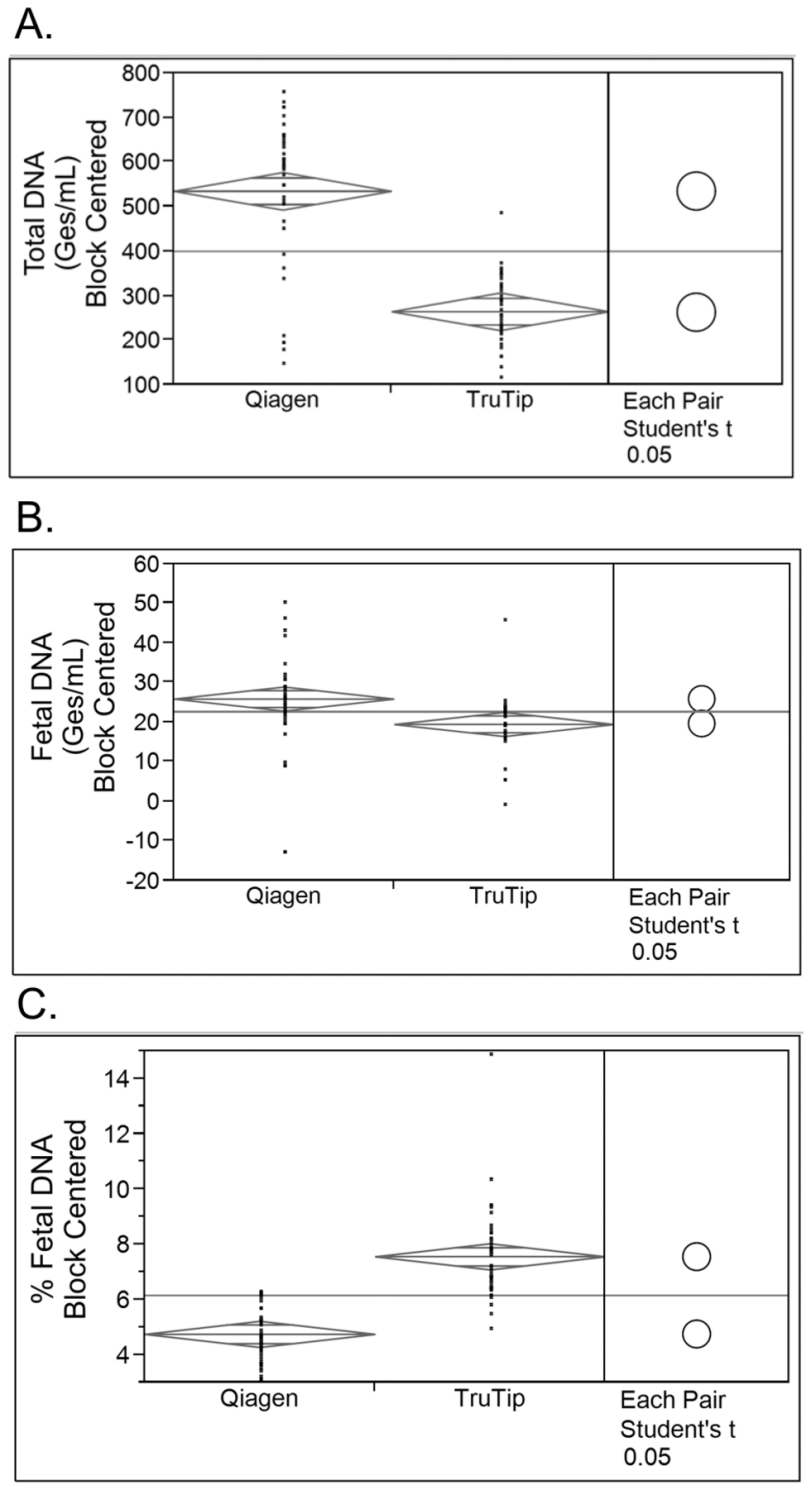
Blood collection tube study qPCR results comparing TruTip and Qiagen extraction methods with Student’s t-test statistical analysis (right side shown as circles) for A) total DNA, B) cffDNA and C) % fetal DNA. Confidence intervals of 95% are represented by the diamond; middle bar equals the mean values with standard deviations above and below.

Extended incubation times prior to plasma processing have resulted in increases in maternal DNA due to lysis of maternal leukocytes. In this study, we examined the effect of incubation time on the extracted material from TruTip and Qiagen methods. Maternal blood specimens were collected and separated into three aliquots for processing based on incubation time at room temperature, with time points of 0, 24, and 48 hrs prior to plasma separation by centrifugation. Samples at each time point were extracted by both TruTip and Qiagen methods to determine whether there was a change in the recovered fetal DNA, total DNA, or percent fetal DNA over time. Recoveries for both extraction methods are shown in Figure 3. The amount of overlap of the circles on the right are a pictorial representation of the statistical significance of the results as calculated using the Student’s t test at α = 0.05. Although results from the two extraction methods differ statistically for all three measurements, total DNA recovery is clearly significantly lower for TruTip processed samples. Because the TruTip procedure *intentionally* reduces maternal DNA of longer length, therefore with lower maternal DNA recovered, the % fetal DNA in the eluent is significantly higher than what is achieved with the Qiagen method by an average of 69% (Figure 3C).

**Figure 4:**
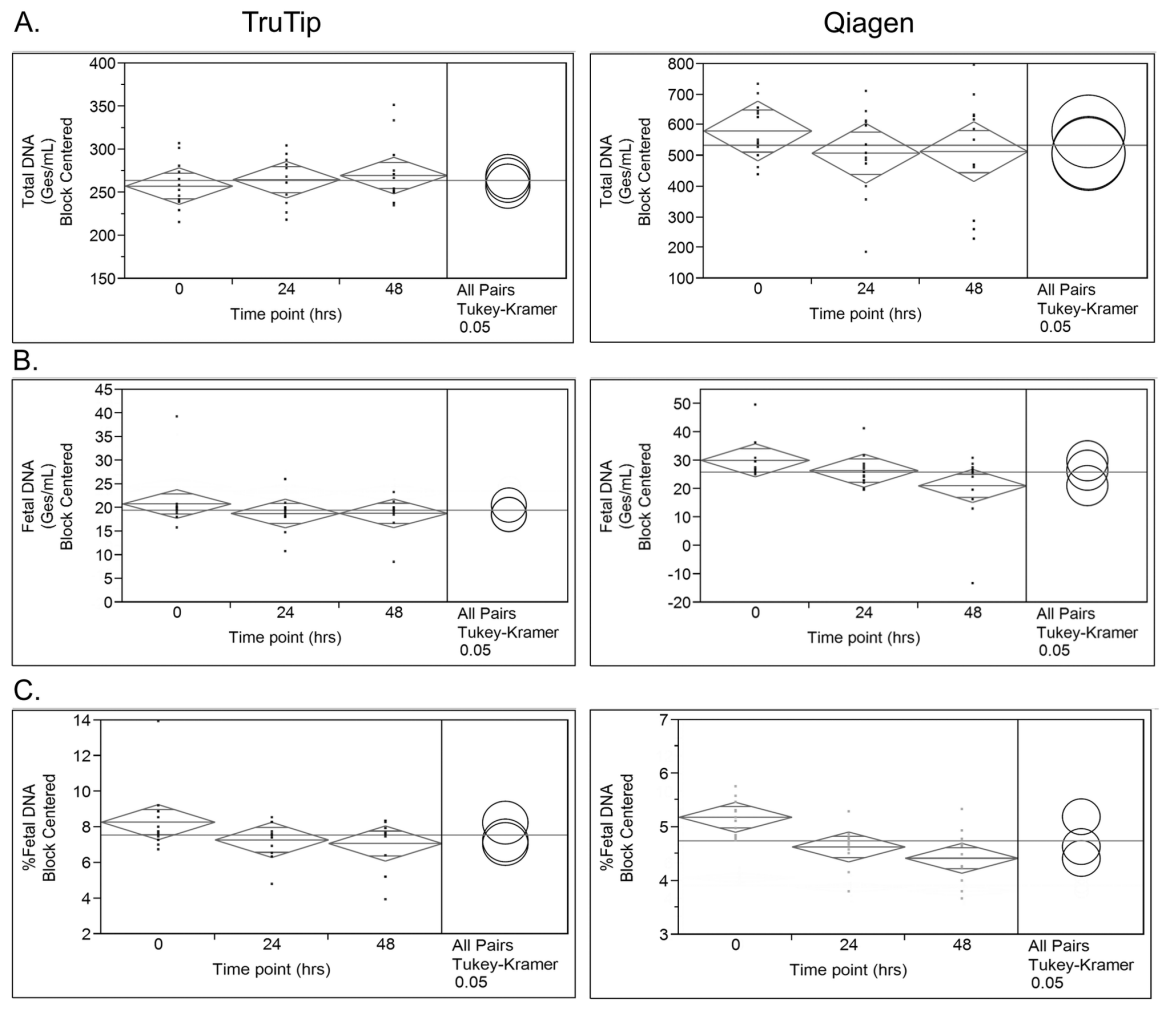
Blood collection tube study qPCR results comparing TruTip (left) and Qiagen (right) extraction methods with Tukey-Kramer statistical analysis (shown to the right as circles) for A) total DNA, B) cffDNA and C) % fetal DNA. Confidence intervals of 95% are represented by the diamond; middle bar equals the mean values with standard deviations above and below.

Figure 4 separates the results by elapsed time points for each extraction method. An all-pairs comparison (Tukey-Kramer HSD) allows for comparison of each data set to the others (e.g. 0 to 24, 0 to 48 and 24 to 48 hrs) and is depicted using overlapping circles to the right of each plotted data set in Figure 4. Though not statistically significant at a confidence level of 95% (p = 0.09), a decrease of 33% in the recovery of fetal DNA using the Qiagen method is observed from 0 to 48 hours (Figure 4B) as compared to only 10% using the TruTip method (p = 0.545). The % fetal DNA recovered with the Qiagen method drops significantly between 0 and 24 hrs, and 0 and 48 hrs of blood storage (p = 0.0210 and 0.0015 respectively), whereas there is no statistically significant decrease in % fetal DNA recovery with the TruTip.

Slower flow through the Qiagen columns or clogging was observed in a number of the maternal samples (6065B-24hr, 6906B-48hr, 1768B-24hr and 6907B- 24hrs, 6066B-48hr, 5656B-48hr, respectively). Most of these samples showed lower yields for both fetal and total DNA compared to TruTip yields. In those where clogging occurred, the filters had to be removed from the vacuum manifold and the protocol completed using the centrifuge along with the typical washing and elution steps.

One anomaly to the data set that does not follow the above trend is sample 6906B. For this sample the TruTip extraction method yielded 14.53% fetal DNA at time zero, dropping to 5.44% and 4.58% at 24 and 48 hours respectively. The Qiagen results for qPCR remained relatively constant for this particular sample (3.51%, 3.41%, 3.33% at 0, 24 and 48 hours, respectively), though consistently lower compared to TruTip. These results suggest that the initial % fetal DNA value of 14.53% for TruTip at time zero is an outlier.

**Figure 5:**
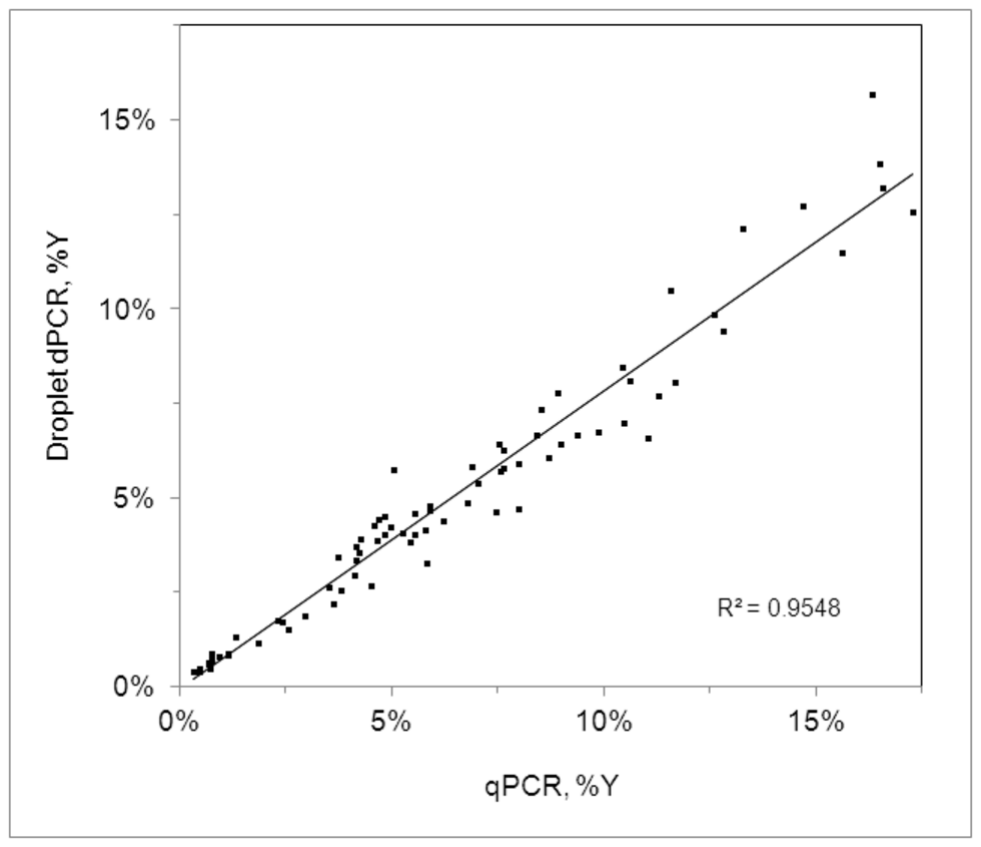
Blood collection tube study results comparing qPCR to ddPCR. Correlation plot of percent fetal, % Y (ddPCR vs. qPCR) with a linear fit.

The extracted samples from the BCT time study were run on both qPCR and ddPCR instruments to compare the accuracy of these two detection methods. Figure 5 shows the correlation between the percent fetal (% Y) values for qPCR and ddPCR. The linear bivariate fit of ddPCR % Y by qPCR % Y demonstrates relatively good concordance between the two methods (~96% - for a perfect fit, every point would lay on the diagonal). Here the data indicate that the two methods correlate better at lower % Y, whereas the qPCR method of detection results in slightly higher % fetal DNA values as compared to ddPCR in the higher ranges (up to 3-4% difference).

## Discussion

In this study we compared extraction of cffDNA from large volumes of maternal plasma using the Akonni TruTip Kit and Qiagen Circulating Nucleic Acid Kit under simulated conditions, as well as in two separate studies involving clinical maternal blood samples. We determined that both methods are able to efficiently isolate DNA over a range of fetal DNA in a background of female DNA relevant to typical concentrations found in early gestation (6-8 weeks). The TruTip kit resulted in a higher % fetal DNA than the Qiagen kit in all but 2 of the 50 samples tested in this study. Though there does not seem to be a reason for these two inconsistencies, both instances occurred in the time point study, sample 5426B at 24 hours and sample 6905B at 48 hours exhibiting a -0.024 and -0.825 difference in % fetal DNA, respectively. In the case of sample 6905B (48hr), the fetal DNA and total DNA recovery from the Qiagen method was significantly lower than that for TruTip (4.2 vs. 8.4 GE/µL for fetal DNA and 98 vs. 246 GE/µL for total DNA, Qiagen and TruTip respectively), indicative of a possible processing issue for the Qiagen sample, though no problems were noted during the extraction. Nevertheless, in both simulated samples and maternal clinical samples, the TruTip method consistently isolated a higher % fetal (or male) DNA through the implementation of the enrichment step, unique to the TruTip protocol. These results suggest that the TruTip binding matrix and buffer component combinations allow the flexibility to selectively bind fragments of different sizes to effectively enrich the amount of fetal DNA present in the sample.

### Workflow Comparison

The Qiagen Circulating Nucleic Acids Kit used a vacuum manifold capable of processing up to 24 samples at a time. Each sample required a single spin column and a large volume plastic adapter to accommodate volumes up to 5 mL of plasma along with the lysis and binding buffers. However, parallel processing of more than a few samples put stress on the vacuum pump, in some cases causing slower, uneven flow through the columns or clogging, as was experienced in at least six of the previously mentioned maternal samples. Though the Qiagen protocol required less hands-on time than the TruTip protocol in the current format, it was overall 30-60 minutes longer and did not include an enrichment step.

The TruTip protocol was similar to Qiagen in its basic biochemistry and number of steps, but very different in relative workflow. The two-step TruTip extraction process included an initial extraction of the total DNA present in the sample using a large volume TruTip. Each sample set-up required an individual pipette, limiting throughput to 1 sample per operator for this initial extraction unless a ring stand and clamps were used to set up 2 to 4 at a time. In the second step, enrichment and concentration of the fetal DNA was performed using a smaller, 1 mL volume TruTip (single or multichannel). For enrichment, the larger fragments of DNA were depleted from the sample by binding them to and rinsing them off of the matrix. After altering the buffer conditions in the sample tubes, a second binding event targeting the smaller fragments was performed with the TruTips, followed by washing and final elution of the remaining DNA fragments. The extra enrichment step resulted in a quantified decrease in maternal DNA in the final isolated sample. Further tuning of this process through optimization of buffer components, elution efficiency, and automation for even higher consistency may improve fetal DNA recovery and further lower the background maternal DNA.

There are two main sources of maternal DNA in plasma. Cell-free circulating maternal DNA is largely fragmented, with some fragmented to the same extent as cell-free fetal DNA circulating in maternal blood, and thus, a complete exclusion of maternal DNA by size separation is not feasible. This portion with size similarity to fetal circulating DNA explains why the effective average enrichment was lower for the clinical samples used in the second two studies compared to the amended samples in the dilution series study (57% and 69% versus 84%, respectively). The second source of maternal DNA is the genomic DNA from peripheral white blood cells lysed during the blood draw, transfer, storage, and plasma separation process. This DNA is much higher molecular weight compared to the fragmented circulating DNA. This longer maternal DNA is what is captured and discarded during the TruTip protocol, resulting in a higher percentage recovery of fetal DNA. Future studies will involve comparing the enriched fetal extracts to the non-enriched extracts to determine the benefit of the decreased maternal DNA in the sample for downstream testing.

The Streck Cell-Free DNA™ BCT tube contains a proprietary preservative to stabilize white blood cells, preventing the release of genomic DNA, and favoring isolation of high-quality cell-free DNA. Previous studies have shown that the use of cell-stabilizing tubes can maintain the same amount of total cell-free DNA over time using droplet digital PCR [4] and qPCR [29] detection methods. These results were confirmed in the current study as shown in Figure 4A, with a relatively stable amount of total DNA recovered at all three time points for each extraction method. Nevertheless, our results indicated a greater stabilization of the % fetal DNA content using TruTip compared to Qiagen. However, unexpectedly, the decrease in % fetal DNA content with the Qiagen kit was caused by a decrease in fetal DNA recovery over time, instead of by an increase in total DNA recovery. This trend was observed in a previous study, for BCT tubes stored at 4° C, but not for those stored at room temperature [29]. However, the decrease in fetal DNA observed with the Qiagen kit was likely due to poor recovery from those samples with previously mentioned processing issues. For TruTip, the decrease of recovered fetal DNA and percent fetal DNA over time was not observed to the same extent, implying that TruTip provides a more stable extraction method when used with Streck tubes.

While the Cell-Free DNA BCT tubes are very effective at stabilizing the unlysed cells and maintaining consistent total cell-free DNA, they are also quite costly (in excess of $10 per tube) and currently only available for research use only. K_2_EDTA tubes, on the other hand, are at least 50-100 times less expensive; however, these tubes result in significant lysis of residual white blood cells over time, greatly inflating the maternal DNA concentration and decreasing the ratio of fetal to maternal DNA in the plasma sample [4]. Future studies will use the TruTip enrichment method with less expensive K_2_EDTA tubes to determine whether this method could stabilize the total DNA recovery and thereby maintain a more constant % fetal DNA over time. Even if recovery is impacted after 24 hours, this would still allow ample time for sample shipment and processing and save money on the total cost of the test. Thus, there is a significant potential benefit to being able to use the less expensive collection tubes in conjunction with the TruTip extraction method.

Finally, the extraction product eluents were amenable to both qPCR and ddPCR methods of detection. Digital PCR has emerged as a more accurate method of quantitation because it is an absolute measurement, thus removing the need for a reference standard curve as for qPCR. Instead, with ddPCR, Poisson distribution statistics are employed to back-calculate the concentration of the target, and allow discrimination of differences as little as 1.5-fold. This level of resolution is valuable for detection of copy number variants and aneuploidies [12,19,27] and has even been shown to detect ≥ 2% higher fetal composition compared to qPCR using microfluidic dPCR [30]. In any case, in our study, no significant difference was found between the two detection methods with respect of extraction performance. Though the % fetal DNA calculations from qPCR data were typically 3-4% higher than those for ddPCR, the observed differences between the extraction methods were consistent.

Though manual methods are presented here, automated solutions are preferable to improve process workflow and sample traceability, and to decrease overall variability in clinical testing situations [31]. Of the two methods evaluated here, the simple pipette-driven technology of TruTip is most amenable to automation on a liquid handling platform. Recently, the automated TruTip method has been described for isolation of genomic DNA from whole blood as well as cell-free DNA [24]. Progress toward this goal was presented in a poster at the American Society for Human Genetics conference in November 2012 [32]. Conversely, the Qiagen method requires more costly instrumentation, including a centrifuge or vacuum manifold that is less consistent when automated due to flow variability and clogging issues similar to those observed in the current study.

In summary, the Akonni TruTip extraction method was shown by real-time qPCR and droplet digital PCR to enrich fetal DNA by selectively depleting longer length maternal DNA, in this manner yielding and overall higher % fetal DNA recovery than the Qiagen isolation method. The TruTip method was also more effectively able to maintain the percent fetal composition over time as compared to the Qiagen system. These results suggest that nucleic acid products isolated using the TruTip extraction process are amenable for use in non-invasive prenatal testing using PCR detection techniques and next generation sequencing (data not shown). The consistent enrichment for fetal DNA observed with the TruTip method offers the potential to significantly improve the detection and subsequent analysis of specific genetic sequences of interest. This method can also be applied to other cell-free DNA target applications including cancer markers and transplant monitoring.

## Supporting Information

Table S1 **Blood collection tube study results.** Table of patient samples with corresponding gestation age, volume of sample extracted, plasma processing time point, qPCR results and calculated percent fetal DNA comparing TruTip (TT) to Qiagen (Q) method. Graphed results of these values are shown in Figures 3 and 4.
(XLSX)
